# Members of the public in the USA, UK, Canada and Australia expressing genetic exceptionalism say they are more willing to donate genomic data

**DOI:** 10.1038/s41431-019-0550-y

**Published:** 2019-11-29

**Authors:** Anna Middleton, Richard Milne, Heidi Howard, Emilia Niemiec, Lauren Robarts, Christine Critchley, Dianne Nicol, Barbara Prainsack, Jerome Atutornu, Danya F. Vears, James Smith, Claire Steed, Paul Bevan, Erick R. Scott, Jason Bobe, Peter Goodhand, Erika Kleiderman, Adrian Thorogood, Katherine I. Morley, on behalf of the Participant Values Work Stream of the Global Alliance for Genomics and Health

**Affiliations:** 1 Society and Ethics Research, Connecting Science, Wellcome Genome Campus, Hinxton, CB10 1SA UK; 2 Faculty of Education, University of Cambridge, Cambridge, UK; 3 Institute of Public Health, University of Cambridge, Cambridge, UK; 4 Centre for Research Ethics and Bioethics, Uppsala University, Uppsala, Sweden; 5 Department of Statistics and Epidemiology, Swinburne University of Technology, Melbourne, & Centre for Law and Genetics, University of Tasmania, Hobart, TAS Australia; 6 Centre for Law and Genetics, University of Tasmania, Hobart, TAS Australia; 7 Department of Political Science, University of Vienna, Vienna, Austria; 8 Department of Global Health & Social Medicine, King’s College London, London, UK; 9 School of Health Sciences, University of Suffolk, Ipswich, UK; 10 Centre for Biomedical Ethics and Law, Department of Public Health and Primary Care, KU Leuven, Leuven, Belgium; 11 Leuven Institute for Human Genomics and Society (LIGAS), KU Leuven, Leuven, Belgium; 12 Melbourne Law School, University of Melbourne, Parkville, VIC Australia; 13 Murdoch Children’s Research Institute, Parkville, VIC Australia; 14 Web Team, Wellcome Sanger Institute, Wellcome Genome Campus, Cambridge, UK; 15 Department of Genetics & Genomic Sciences, Icahn School of Medicine at Mount Sinai, New York, NY USA; 16 Institute for Next Generation Healthcare, Icahn School of Medicine at Mount Sinai, New York, NY USA; 17 Ontario Institute for Cancer Research, MaRS Centre, Toronto, ON Canada; 18 Centre of Genomics and Policy, McGill University, Montreal, QC Canada; 19 Institute of Psychiatry, Psychology, and Neuroscience, King’s College London, London, UK; 20 Centre for Epidemiology and Biostatistics, Melbourne School of Global and Population Health, University of Melbourne, Melbourne, VIC Australia; 21 RAND Europe, Cambridge, UK

**Keywords:** Ethics, Social sciences

## Abstract

Public acceptance is critical for sharing of genomic data at scale. This paper examines how acceptance of data sharing pertains to the perceived similarities and differences between DNA and other forms of personal data. It explores the perceptions of representative publics from the USA, Canada, the UK and Australia (*n* = 8967) towards the donation of DNA and health data. Fifty-two percent of this public held ‘exceptionalist’ views about genetics (i.e., believed DNA is different or ‘special’ compared to other types of medical information). This group was more likely to be familiar with or have had personal experience with genomics and to perceive DNA information as having personal as well as clinical and scientific value. Those with personal experience with genetics *and* genetic exceptionalist views were nearly six times more likely to be willing to donate their anonymous DNA and medical information for research than other respondents. Perceived harms from re-identification did not appear to dissuade publics from being willing to participate in research. The interplay between exceptionalist views about genetics and the personal, scientific and clinical value attributed to data would be a valuable focus for future research.

## Introduction

Genomic medicine is being integrated into healthcare in many countries as an emerging basis of clinical care, public health and disease prediction [1]. The collection and sharing of genomic data is fundamental to this goal, driving research and clinical applications, underpinning the ability to do accurate variant interpretation [2]. Researchers have called for global genomic data sharing, enabling databases to be connected internationally across geographical, legal and policy borders, for databases to be more easily accessible and to ensure that they better represent the populations having genomic testing [1, 3, 4]. The collection of genomic information at scale is thus increasing, and major projects are underway to link genetic and health data of millions of citizens [1, 5].

Data sharing presents a number of challenges, notably related to data confidentiality, risk of discrimination, and the need for appropriate governance structures [6]. However, one important consideration in discussions of data sharing pertains to the perceived similarities and differences between DNA and other forms of personal data. In this paper, we consider the relationship between perceptions of ‘genetic exceptionalism’ and the willingness to donate and share DNA information.

The concept of ‘genetic exceptionalism’ suggests that genetic information has different properties than other types of medical information, i.e., it is ‘special’ because it is uniquely identifying, directly links us to our relatives or can provide information about our past, present and future health [7]. Such considerations have been emphasised by some commentators and inform policy such as the USA’s Genetic Information Nondiscrimination Act, which regards genetic data as unique and meriting increased protection [7, 8]. Others contend that features of genetic information are shared with characteristics such as socio-economic status, HIV status or family history [9]. They suggest that DNA can be seen as distinct and as warranting a special respect for privacy, without legal or regulatory protection beyond that accorded to other sensitive information [10].

Genomic and health data come from health services, biobanks, research projects and donated blood [11, 12]. They originate from individuals who have a right to have a say in how this data will be used; at the very least, they should have consented for their de-identified data to be shared with researchers and clinicians [13]. Furthermore, even if an individual has not yet encountered an opportunity to donate their data they are increasingly likely to be genetically related to someone who has. The decisions that one person makes thus have the potential to be relevant to relatives. To protect the rights of patients and research participants and to respect their expectations and values related to data use, it is important to incorporate the views of broader publics as stakeholders in decision making. It is thus timely to explore global public perceptions of issues surrounding the use of de-identified DNA information within the contexts of genomic research and clinical practice [14].

Our research examined whether ‘genetic exceptionalism’, defined as the belief that DNA information is different from other forms of medical information, is associated with the willingness or unwillingness to donate data. We further examined whether it is associated with different perceptions of potential harms arising from sharing DNA information. Research has identified the importance of concerns about discrimination and the privacy of DNA information in shaping decisions about research participation [15–17]. What has yet to be studied is the extent to which willingness to donate and share DNA information and perception of harms among the general public is shaped by ‘exceptionalist’ perceptions of genetic information. While exceptionalism might increase concerns about privacy, it also suggests the potentially distinctive value of genetic information.

In this paper, we describe the perceptions of English-speaking respondents from the UK, USA, Australia and Canada to the ‘Your DNA, Your Say’ (YDYS) study. YDYS aims to examine public perceptions of genomic data sharing across international and language borders and involve, where practically possible, representative samples of publics. Although important empirical work has already been conducted in this space, particularly in the context of biobanking, there remains limited published literature on global public attitudes towards personal donation of DNA information [16, 18]. The survey is a global project that has been translated into multiple languages. Once global recruitment is complete, we will publish separately on a between-country meta-analysis of attitudes.

## Materials and methods

Details of the study design, methodology, recruitment and data collection are published separately, as is a review of the context and background of this project [19–21].

### Sample

Using the market research company Dynata to invite public audiences to participate, we collected surveys from publics in the USA, Canada, UK and Australia (*n* = 8967). Participants were paid a small financial reward (<£1) for participating. Owing to the nature of recruitment, there are no details on non-response rate. Dynata invited represenative public from each country to participate and we demonstrate how these are matched in terms of age and gender.

### Measures

Our cross-sectional, exploratory online survey can be accessed at YourDNAYourSay.org. It contains 29 questions and piloting showed it took 15–20 min to complete.

#### Sociodemographic information

Information about age was collected in 10-year categories from age 16 onwards; due to the low number of responses in younger and older age categories these were collapsed to ‘30 years and under’ and ‘60 years and older’. Whether participants had children was determined by the question ‘Do you have children?’ Participants were asked to answer ‘Yes’ or ‘No’ and were not asked to specify whether they were their biological children. Relationship status was collected as ‘Divorced’, ‘Separated’, ‘Single’, ‘Widowed’, ‘Married/civil partnership/living together’; all categories apart from the latter were collapsed for analyses.

We piloted how to collect ethnicity data, starting with the categories provided in the UK Census survey and adapting these based on feedback from pilot participants. The resultant ethnicity question in the final survey asked participants to self-identify as: White; Afro-European/African American, Black; Hispanic; South Asian, Indian, Pakistani; East Asian Chinese, Japanese; Arabic, Central Asian; Other. Participants could choose not to answer this question. Owing to the low number of participants who self-identified as a member of a group other than ‘White’ (<10% of the sample for each country), these were collapsed into a single ‘Non-White’ category for analysis.

Education level was categorised as ‘Tertiary’, ‘Secondary’, ‘Primary’ or ‘Other’ based on structured and free text descriptions of educational qualifications and collapsed to a binary indicator of tertiary education for multivariable analyses. Religiosity was determined by response to the question ‘Independent of whether you attend religious services or not, would you say you are…?’ with options ‘A religious person’ or ‘Not a religious person’.

#### Genetics experience

Genetics experience was derived from two questions: ‘Are you familiar with DNA, genetics or genomics?’ and ‘I’m familiar through my work, personal interests or family/medical history’. Participants were categorised as having ‘Personal’ experience of genetics if they said they were familiar and that familiarity was due to either having a genetic condition in their family, or through their work (e.g., genetic health professional or genetic researcher). Participants without this experience were categorised as ‘Familiar’ or ‘Unfamiliar’ based on their response to the first question.

#### View of genetic information

Participants were asked if they believed DNA information was the same as other sorts of medical information. The question used was:

Some people think DNA information is the same as any other medical information, like blood pressure or blood sugar levels. Others think DNA information is special, for example, because it tells us how we are related to other people. What do you think?

- For me, DNA information is different to other medical information.
- For me, DNA information is the same as other medical information.
- I’m not sure.

Those who answered that DNA information was different were categorised as having ‘genetic exceptionalist views’. As we were primarily interested in people who had clear exceptionalist views, responses indicating that the participant was unsure or did not think DNA information is the same were combined.

#### Potential for harm

Participants were asked a single question regarding harms associated with linking personally identifying information to their DNA data: ‘If someone linked your name, address and phone number to it, do you think you could be harmed in any way from this?’. Response options were ‘Yes’, ‘No’, ‘I’m not sure’ with the latter two categories collapsed for analysis. As we were primarily interested in whether people had a clear understanding of the harms, the ‘No’ and ‘Unsure’ categories were collapsed for analysis.

#### Concerns about specific harms

Participants were presented with a list of theoretical harms that could occur in relation to DNA information and asked to indicate which were the three that concerned them most. The list of theoretical harms presented to participants was:

- My friends potentially knowing something about me that I hadn’t chosen to tell them.
- My family potentially knowing something about me that I hadn’t chosen to tell them.
- My government potentially knowing something about me that I hadn’t chosen to tell them.
- Police potentially knowing something about me that I hadn’t chosen to tell them.
- Marketing companies targeting me to sell me products
- Being stigmatised and labelled in some way online.
- Being cloned.
- My DNA being copied and then planted at the scene of a crime.
- Health or life insurance companies using the information to discriminate against me.
- Employers using the information to discriminate against me.
- Upsetting my genetic relatives.
- Ethnic identification and racial discrimination.

#### Donating DNA and medical information

Throughout the survey, participants were asked whether they would donate their ‘anonymous’ DNA and medical information for use by others in research [1]^1^. Participants were asked whether they would donate DNA and medical information for use by (a) medical doctors; (b) non-profit researchers; (c) for-profit researchers. Participants were classified as willing to donate if they answered yes to at least one of these questions, and as unwilling if they answered no to all three. In terms of influences on donating, participants were asked ‘Would being offered a DNA readout influence your decision to donate?’ and ‘Would you be more comfortable donating your DNA and/or medical information if you knew there was a law in place to protect against being exploited?’. Finally, participants were asked ‘Would you allow someone else, such as an ethics committee or custodian, to decide on your behalf which researchers and studies could use your DNA and/or medical information?’.

### Statistical analysis

Sample characteristics were summarised using standard descriptive statistics, with differences between those with and without exceptionalist views evaluated using chi-squared tests. Multivariable analyses were conducted using multi-level binary logistic regression models to allow for clustering of participants within countries, and estimation of the variability in the outcome variables based on country of residence. The models provide odds ratio (OR) estimates for the association between perspective on DNA information and either perspective on harms or willingness to donate, holding country of residence constant [22, 23].

#### Multivariable analyses

The multivariable analysis investigated the association between perceptions of DNA information and (i) perceptions of harms associated with linking DNA to other personal information; (ii) willingness to donate DNA for research. Familiarity with genetics, age, gender, ethnicity, marital status, having children, education level, and religiosity have previously been associated with perceptions of genetics and were included as covariates [24–26].

Models included a random country-level intercept to allow for between-country variation in perspectives on harms associated with linking DNA, and willingness to donate DNA for research. The effect of country of residence was quantified using the intraclass correlation and median odds ratio [27]. Perspective on DNA information was initially modelled as a fixed effect; random slope models were then fitted to evaluate whether the associations varied by country of residence. Familiarity with genetics, age, gender, ethnicity, marital status, having children, education level, and religiosity were included as fixed effects. As we anticipated that the relationship between exceptionalism and the outcome variables would be influenced by familiarity with genetics, an interaction term between perspective on DNA information and familiarity with genetics was also included.

Models were fitted via maximum likelihood with difference in model fit evaluated using likelihood ratio chi-squared tests and akaike information criterion (AIC) where appropriate [28]. As the alternative hypotheses regarding variances are technically one-sided, having the *p*-value for these tests has been suggested; we report the standard *p*-values but consider this modification when interpreting results [29]. Complete-case analyses were conducted in R version 3.3.1 [30] using the lme4 package for multi-level models, with 95% confidence intervals (CI) for the final model parameter estimates obtained using bootstrapping with 1000 replicates per model [31].

## Results

### Sample description

Comparison with most recent census data from each country shows the sample is representative for gender, but with a slight over-sampling of younger age groups and under-sampling of those over 60 years of age (Table S1). There was some variation between those who did and did not have genetic exceptionalist views for all sociodemographic variables with the exception of gender and ethnicity (Table 1); however, there were minimal between-country differences for this variable.

**Table 1:** Sample characteristics by perspective on seeing DNA information as the same/unsure or different to medical information (‘genetic exceptionalist’ views) (*N* indicates count; % indicates percentage)

		Total (*n* = 8965)		Same/unsure (*n* = 4337)		Different (*n* = 4628)		
Variable	Categories	*N*	%	*N*	%	*N*	%	*p*
Genetics knowledge	Unfamiliar	5004	55.8	2801	64.6	2203	47.6	≪0.001
	Familiar	2787	31.1	1157	26.7	1630	35.2	
	Personal	1173	13.1	378	8.7	795	17.2	
	Missing	1	0	1	0	0	0	
Age	30 and under	2091	23.3	993	22.9	1098	23.7	≪0.001
	31–40	2047	22.8	959	22.1	1088	23.5	
	41–50	1569	17.5	823	19	746	16.1	
	51–60	1588	17.7	824	19	764	16.5	
	Over 60	1664	18.6	734	16.9	930	20.1	
	Missing	6	0.1	4	0.1	2	0	
Gender	Female	4328	48.3	2114	48.7	2214	47.8	0.255
	Male	4574	51	2178	50.2	2396	51.8	
	Missing	63	0.7	45	1	18	0.4	
Children	No	3696	41.2	1925	44.4	1771	38.3	≪0.001
	Yes	5112	57	2324	53.6	2788	60.2	
	Missing	157	1.8	88	2	69	1.5	
Education	Tertiary	5173	57.7	2375	54.8	2798	60.5	≪0.001
	Secondary	3009	33.6	1520	35	1489	32.2	
	Primary	551	6.1	309	7.1	242	5.2	
	Other	224	2.5	128	3	96	2.1	
	Missing	8	0.1	5	0.1	3	0.1	
Country	United Kingdom	3316	37	1656	38.2	1660	35.9	≪0.001
	United States	1992	22.2	862	19.9	1130	24.4	
	Canada	2255	25.2	1112	25.6	1143	24.7	
	Australia	1402	15.6	707	16.3	695	15	
Ethnicity	White	7539	84.1	3599	83	3940	85.1	0.049
	Other	1315	14.7	667	15.4	648	14	
	Missing	111	1.2	71	1.6	40	0.9	
Religiosity	Not a religious person	5609	62.6	2897	66.8	2712	58.6	≪0.001
	A religious person	3349	37.4	1435	33.1	1914	41.4	
	Missing	7	0.1	5	0.1	2	0	
Relationship	Married/civil partnership/living together	5565	62.1	2628	60.6	2937	63.5	0.006
	Divorced/Single/Widowed	3393	37.8	1704	39.3	1689	36.5	
	Missing	7	0.1	5	0.1	2	0	

*p*-value shown for chi-squared test between perspectve of DNA and each variable

Participants with genetic exceptionalist views (~52% of survey respondents) were more likely to report: familiarity with genetics (35.2% versus 26.7%); personal experience with genetics (17.2% versus 8.7%); having children (60.2% versus 53.6%); tertiary-level education (60.5% versus 54.8%); and being religious (41.4% versus 33.1%). We adjusted for all potential confounding effects in the multivariable modelling.

### Associations between potential harms, willingness to donate and genetic exceptionalism

**Fig. 1:**
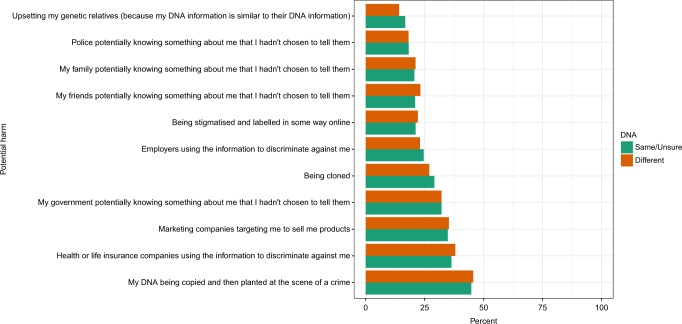
Association between perspective on seeing DNA information as the same/unsure or different to medical information (‘genetic exceptionalist views’) and perceived harms that could arise if a person was identified from their DNA information

Participants holding genetic exceptionalist views were substantially more likely to think that linking personally identifying information to their DNA information could potentially harm them in some way (49.5% compared to 35.2%; see Table 2). However, concerns about specific harms did not vary significantly between the two groups. Of the 11 potential harms participants were asked to rate, the most frequently identified by both those with and without genetic exceptionalist views was that related to ‘My DNA being copied and then planted at the scene of a crime’ (included in the top three concerns by 45.2% of the sample). The next most frequently identified were ‘Health or life insurance companies using the information to discriminate against me’ (37.2%) and ‘Marketing companies targeting me to sell me products’ (35%) (Fig. 1 shows these results broken down by views on genetic exceptionalism).

**Table 2:** Bivariate associations between perspective on seeing DNA information as the same/unsure or different to medical information (‘genetic exceptionalist’ views) and views on potential harms, and issues around donation of DNA for research (*N* indicates count; % indicates percentage)

		Total		Same/unsure		Different		
Variable	Categories	*N*	%	*N*	%	*N*	%	*p*
Harms from linking DNA and personal info	No	5145	57.4	2808	64.7	2337	50.5	≪0.001
	Yes	3817	42.6	1526	35.2	2291	49.5	
	Missing	3	0	3	0.1	0	0	
Would donate DNA/medical info	No/unsure to all	3901	43.5	2294	52.9	1607	34.7	≪0.001
	Yes to all/some	5064	56.5	2043	47.1	3021	65.3	
Donation influenced by getting DNA readout	Would not donate/unsure	4136	46.1	2455	56.6	1681	36.3	≪0.001
	Donate regardless	1176	13.1	569	13.1	607	13.1	
	Yes	3651	40.7	1311	30.2	2340	50.6	
	Missing	2	0	2	0	0	0	
Donation influenced by legal protection	No/unsure	3959	44.2	2288	52.8	1671	36.1	≪0.001
	Yes	5001	55.8	2046	47.2	2955	63.9	
	Missing	5	0.1	3	0.1	2	0	
Would allow REC to make decisions	Would not donate/unsure	4857	54.2	2722	62.8	2135	46.1	≪0.001
	No	2545	28.4	1104	25.5	1441	31.1	
	Yes	1558	17.4	507	11.7	1051	22.7	
	Missing	5	0.1	4	0.1	1	0	

*p*-value shown for chi-squared test between perspective of DNA and each variable (excluding missing data)

Despite being more likely to identify the possibility of harm, participants with genetic exceptionalist views were substantially more likely to accept that their ‘anonymous’ DNA and/or medical information should be donated for research purposes (65.3% versus 47.1%, see Table 2). Their decision on whether to contribute their data to research was also more likely to be influenced by the prospect of receiving a readout of their own DNA upon sharing their DNA (50.6% versus 30.2%) and by knowing there are legal protections in place to prevent exploitation (63.9% versus 47.2%). Irrespective of participants’ view of genetic exceptionalism, only a minority (17.4%) were comfortable with allowing a research ethics committee to make decisions on their behalf regarding research use of their DNA and/or medical information. However, those who had genetic exceptionalist views were more likely to be comfortable with this (22.7% versus 11.7%).

### Multivariable associations with perceived harm of linking DNA information

We first fitted ‘empty’ multi-level models to investigate how much variability in participant views of whether linking DNA and personal information could harm them could be explained by participant country of residence [32]. The likelihood ratio test comparing fixed effects and random intercept models indicated that there was significant variance explained by the between-country effect on perceived harm ($\chi_{1}^{2}$ = 54.3; *p* < 0.0001). However, the intraclass correlations and median ORs were small; the percentage of variance in perceived harm explained by country of residence was only 0.8% and the median OR was 1.09.

Allowing the effect of participants’ views of the difference between DNA and other medical information to vary by country of residence did not significantly improve model fit ($\chi_{2}^{2}$ = 0.49; *p* = 0.78), indicating the strength of relationship between exceptionalism and perceived harm was similar across countries. Consequently this variable was included as a fixed effect in the full multivariable model. We tested the importance of the interaction between perception of DNA information and familiarity of genetics, and found moderate evidence to support it for this outcome variable ($\chi_{2}^{2}$ = 5.64; *p* = 0.06; AIC 11,464 versus 11,463). Results from the full model are shown in Table 3.

**Table 3:** Results from the multivariable multi-level model for perceived harms from linking DNA and personally identifying information, and for willingness to donate DNA and medical information for research

		Perceived harms (*N* = 8704)			Donating for research (*N* = 8704)		
Variable	Categories	Beta	95% CI	*p*	Beta	95% CI	*p*
*Fixed effects*							
DNA status	Same as other medical information	Ref.			Ref.		
	Different	0.57	0.44 to 0.68	≪0.001	0.69	0.57 to 0.8	≪0.001
Genetics experience	Unfamiliar	Ref.			Ref.		
	Familiar	0.67	0.51 to 0.81	≪0.001	0.67	0.53 to 0.82	≪0.001
	Personal	0.47	0.22 to 0.7	≪0.001	1.28	1.03 to 1.54	≪0.001
Interaction	Different × familiar	−0.23	−0.43 to −0.02	0.022	−0.24	−0.44 to −0.05	0.016
	Different × personal	0.00	−0.29 to 0.32	0.99	−0.23	−0.56 to 0.11	0.15
Age	50 and older	Ref.			Ref.		
	31−50	−0.11	−0.22 to −0.01	0.03	0.09	−0.02 to 0.2	0.07
	30 and younger	−0.13	−0.25 to 0.01	0.05	0.49	0.36 to 0.62	≪0.001
Gender	Female	Ref.			Ref.		
	Male	0.07	−0.01 to 0.16	0.11	0.08	0 to 0.19	0.03
Children	No	Ref.			Ref.		
	Yes	−0.06	−0.15 to 0.06	0.30	0.15	0.05 to 0.26	0.007
Tertiary education	Yes	Ref.			Ref.		
	No	−0.26	−0.34 to −0.17	≪0.001	−0.24	−0.37 to −0.19	≪0.001
Ethnicity	White	Ref.			Ref.		
	Other	0.04	−0.09 to 0.18	0.50	−0.34	−0.47 to −0.21	≪0.001
Religiosity	Not a religious person	Ref.			Ref.		
	A religious person	0.17	0.07 to 0.26	≪0.001	0.12	0.03 to 0.22	0.01
Relationship status	Married/civil partnership/living together	Ref.			Ref.		
	Divorced/single/widowed	0.01	−0.11 to 0.12	0.89	−0.06	−0.16 to 0.04	0.26
*Random effects*							
Intercept variance		0.02	0 to 0.04		0	0 to 0.003	

Based on the full multivariable model (Tables 3 and  4), participants who had genetic exceptionalist views had greater odds of thinking that linking their DNA information to identifying personal details could potentially cause them harm, compared to others with the same level of genetics familiarity [OR 1.76 (95% CI: 1.56–1.98), OR 1.40 (1.19–1.65), OR 1.76 (1.35–2.31)] for ‘unfamiliar’, ‘familiar’, and ‘personal’ strata, respectively). Those who had genetic exceptionalist views *and* were familiar with genetics had greater odds of thinking this could potentially cause them harm compared to those who did not view DNA as different and were unfamiliar with genetics (OR 2.73, 95% CI 2.38–3.13). However, there was minimal difference between those who were familiar with genetics and those who had personal experience through work or a family history of an inherited condition (OR 2.81, 95% CI 2.36–3.35 for the latter).

**Table 4:** Odds ratios for interaction between genetics experience and perspective on DNA information (‘genetic exceptionalist’ views) for (i) thinking that linking DNA and personally identifying could cause potential harm and (ii) willingness to donate DNA for medical research (derived from full multivariable model)

Outcome	Genetics experience	Perspective on DNA data		DNA perspective within genetics experience
		Same/unsure	Different	
Perceived harms	Unfamiliar	Ref.	1.76 (1.56 to 1.98)	1.76 (1.56 to 1.98)
	Familiar	1.95 (1.67 to 2.25)	2.73 (2.38 to 3.13)	1.40 (1.19 to 1.65)
	Personal	1.60 (1.25 to 2.02)	2.81 (2.36 to 3.35)	1.76 (1.35 to 2.31)
Willingness to donate	Unfamiliar	Ref.	1.98 (1.78 to 2.24)	1.98 (1.78 to 2.24)
	Familiar	1.96 (1.70 to 2.28)	3.05 (2.66 to 3.49)	1.55 (1.32 to 1.83)
	Personal	3.58 (2.81 to 4.65)	5.66 (4.64 to 6.91)	1.58 (1.18 to 2.12)

### Multivariable associations with donating DNA information for research

The likelihood ratio test comparing fixed effects and random intercept models did not indicate that substantial variance was explained by the between-country effect on willingness to donate DNA and/or medical information for research ($\chi_{1}^{2}$ = 3.18; *p* = 0.075) and thus a standard logistic regression model could feasibly be fitted. We retained the multi-level model specification to ensure we adequately accounted for between-country differences. Allowing the effect of participant view of the difference between DNA and other medical information to vary by country of residence did not significantly improve model fit ($\chi_{2}^{2}$ = 2.69; *p* = 0.26) and this was, therefore, included as a fixed effect in the full multivariable model. We tested the interaction between willingness to donate DNA and/or medical information and familiarity with genetics and found evidence to support it for this outcome variable ($\chi_{2}^{2}$ = 6.65; *p* = 0.036; AIC 11,171 versus 11,168). Results are shown in Table 3.

In the full multivariable model (Tables 3 and  4), participants who had genetic exceptionalist views had greater odds of being willing to donate their DNA compared to others with the same level of genetics familiarity [OR 1.98 (95% CI: 1.78–2.24), OR 1.55 (1.32–1.83), OR 1.58 (1.18–2.12) for ‘unfamiliar’, ‘familiar’, and ‘personal’ strata, respectively]. Those who had personal experience of genetics *and* genetic exceptionalist views had the greatest odds of being willing to donate, compared to those who were unfamiliar with genetics *and* did not have a genetic exceptionalist view (OR 5.66, 95% CI 4.64–6.91). Those with personal experience of genetics also had greater odds of being willing to donate, even if they did not have genetic exceptionalist views (OR 3.58, 95% CI 2.81–4.65). However, those who were familiar with genetics *and* had genetic exceptionalist views had similar odds of being willing to donate (OR 3.05, 95% CI 2.66–3.49).

## Discussion

In this age and gender-matched sample from the USA, Canada, the UK and Australia, participants who held genetic exceptionalist views were more likely to be familiar with or have had personal experience with genomics. They were also the most likely to say they were willing to donate their ‘anonymous’ DNA and medical information to research, despite also being the most likely to understand that linking DNA information to personal information had the *potential* to cause harm. Thus, while they identified the possibility of harm if they were re-identified from their data, they would still donate, presumably because they accepted the benefits outweighed the perceived harms. This is concordant with the findings of previous research on biobanking and the donation of DNA information for research [33]. In other words, those interested in data donation appear aware of both what genetic information is and its value for research and genomic medicine.

Participants with genetic exceptionalist views and self-reported familiarity with genomics had the greatest odds (nearly 6 times greater) of being willing to donate their DNA information (compared to those who were unfamiliar with genomics and did not have exceptionalist views). Such willingness among those with personal familiarity has been seen in patient groups who want their data to be put to good use for future disease prevention, or to help future generations within their own family [34, 35]. Many of those most willing to donate were also professionals working in the genomics field. This is in agreement with our previous research on the liberal attitudes of scientists to participate in genomic research [36].

The perceived harms that participants associated with being identified from one’s DNA information reflect the influence of popular culture. After secondary education, most non-experts are thought to absorb science information and knowledge from the media [37]. For example, TV shows such as *CSI* (American television programme, made 2000–2015) with 30–50 million viewers worldwide function as a resource for quasi-scientific information about genetics [38]. Media representations play an important role in shaping public familiarity with science, including framing or priming conversations about DNA [39]. The identification by participants of concerns about their DNA being copied and then planted at the scene of a crime fits with this picture. What we are not able to conclude is whether participants have a genuine concern about this, or whether it is made salient and plausible by exposure to popular media.

Risks associated with data sharing may be seen as less concrete and more future-oriented than benefits [18]. However, the relevance of perceived ‘harms’ as actual ‘risks’ needs to be understood in relation to their probability of occurring [40]. Public engagement that discusses the potential risks or harms of linking DNA information to personal information, however small or unlikely, may therefore not adversely affect the willingness of people to donate their data. Indeed, engagement activities that explain the distinctive features of DNA data may in fact support engagement with research.

Those with exceptionalist views were more likely to make decisions about donation based on whether they could obtain a copy of their own raw data (a ‘DNA readout’ or raw sequence data) in return and if they were aware of any clear legal sanctions in place to protect against exploitation. This reflects the perceived personal and economic, as well as clinical and scientific, value of DNA information. This group was more likely to allow research ethics committees (RECs) to make decisions about data sharing on their behalf. These findings suggest the potential value of returning results to this population, but also the corollary, that receiving sequence data may be less appealing to those who do not regard it as something special or distinctive. They also emphasise the importance of transparent processes for decision making around data sharing, and communication about the sanctions consequent upon data misuse.

### Limitations

Exploratory online surveys have important limitations in that they capture perceptions about intended behaviour at a single time point. While intentions are one potential predictor of behaviour, further work is needed to document what people actually do when faced with opportunities to donate data [41]. Generic limitations of the study and online survey design have been published separately [19]. Our findings should not be extrapolated to indicate views of all people from the USA, Canada, the UK and Australia, particularly the older population who may differ in respect of their willingness to donate and beliefs about the uniqueness of genetic information.

## Conclusion

Big data and genomics now go hand in hand, and it is time to bring broader publics into conversation about their willingness to donate their data to be accessed and shared to enable the potential of genomic medicine to be fully realised. To support this, we have explored ‘representative’ English-speaking public perceptions of genomic data donation and some of the characteristics of those reportedly willing to donate their data for use in the research endeavour.

There appear obvious factors that may support and encourage data donation and sharing, such as offering to return results in some form, providing clear information about legal protections, and engagement that addresses the distinctive characteristics of genetic information. The potential of these approaches should be explored and evaluated. However, it is not sufficient to ‘educate’ people about genomics—familiarity does not necessarily equate factual knowledge. Rather, we must work to understand *what* people need to know and *how* to make the subject resonate, so that genomics becomes a social and sociable concept and that citizens can feel comfortable having a basic conversation about its benefits and limitations.

Finally, we found that despite believing that there were potential specific risks from re-identification, these alone did not appear to dissuade publics from being willing to participate in research. Thus, for some, it appears that DNA has sufficient value to warrant donation for research. Exploring the interplay between exceptionalist views and the personal, scientific and clinical value attributed to data would be a fruitful focus for future research.

## Supplementary information

Supplementary material

## Footnotes

1 Within the survey glossary we explained ‘anonymous’ in more detail: ‘Anonymous: removal of personal information such as name and date of birth. It is questionable as to whether DNA information can ever be truly anonymous as our DNA code is unique to us and thus, in itself, could be used to identify us. However, in the circumstances we are exploring here, by making DNA and medical information ‘anonymous’, we mean detaching personal identifiers from it.’ What we are actually meaning here is ‘de-identified’ but within the pilot work for the survey we discovered that public participants did not naturally understand this term and ‘anonymous’ was more easily understood, thus we added the glossary definition within the survey itself, to explain this in more detail.
