# Supplementary material for: Members of the public in the USA, UK, Canada and Australia expressing genetic exceptionalism say they are more willing to donate genomic data
Source: Eur J Hum Genet. 2019 Nov 29;28(4):424–34. doi: 10.1038/s41431-019-0550-y (PMC7080803; doi:10.1038/s41431-019-0550-y)
Supplement: Supplementary file 1 — Supplementary material [file 41431_2019_550_MOESM1_ESM.docx]

Members of the public in the USA, UK, Canada and Australia expressing genetic exceptionalism say they are more willing to donate their genomic data

# Supplementary Appendix

# Comparison with national census data

The YDYS sample was compared with the most recent available census data from each country:

- UK (2011 data; www.ons.gov.uk/census/2011census [accessed 13/09/2017])
- USA (2015 data; www.census.gov [accessed 13/09/2017])
- Canada (2016; <http://www12.statcan.gc.ca/census-recensement/index-eng.cfm> [accessed 13/09/2017])
- Australia (2016; <http://www.abs.gov.au/websitedbs/censushome.nsf/home/2016> [accessed 13/09/2017])

Based on comparison with the census data (Supplementary Table S1), YDYS participants aged over 60 were under-sampled from the UK, USA, and Canada, while younger groups were over-sampled, which is likely to be due to use of online recruitment and survey administration.^1,2,3^ The proportion of women from each country matched the census data, with the exception of the Canadian sample for which fewer women were recruited than expected (41.5% of sample compared to 51.3% for census).

***Table S1:*** *Comparison of YDYS samples with most recent census data from each country: proportion*

*of sample in age and gender categories by country of residence.*

| Variable | Categories | UK | | USA | | Canada | | Australia | |
| --- | --- | --- | --- | --- | --- | --- | --- | --- | --- |
|  |  | YDYS | Census | YDYS | Census | YDYS | Census | YDYS | Census |
| Age | 30 and under | 27 | 24.8 | 17.6 | 25.5 | 25.8 | 22.4 | 21.1 | 18 |
|  | 31-40 | 20.9 | 16.4 | 30.3 | 16.2 | 22.3 | 15.8 | 20.4 | 19 |
|  | 41-50 | 17.9 | 17.9 | 15.8 | 15.8 | 18.3 | 15.8 | 18 | 18 |
|  | 51-60 | 17 | 14.6 | 20 | 16.8 | 16.5 | 18.1 | 16.4 | 17 |
|  | Over 60 | 17.2 | 26.3 | 16.2 | 25.7 | 16.9 | 28.1 | 24.2 | 28.2 |
| Gender | Female | 50.3 | 50.8 | 51.3 | 50.7 | 41.5 | 51.3 | 51.8 | 51 |
|  | Male | 49 | 49.2 | 48.1 | 49.3 | 57.1 | 48.7 | 47.6 | 49 |

# Data cleaning

For a survey focused on personal opinions, and a subject matter that is likely to be unfamiliar to research participants, checking internal consistency of participant answers is difficult, but we did so where possible. We focused on responses to two different sets of questions that elicited views on whether participants would be willing to donate their DNA and medical information for research conducted by medical doctors, non-profit researchers, and for-profit researchers. For early respondents in the UK sample, this first involved correcting a data collection inconsistency due to an error in the survey coding that allowed participants who had said they would not donate their DNA and medical information to access question subsections regarding who they would donate to. This coding error was subsequently corrected, but the data for these early participants was cleaned so that responses to the sub-questions were removed for those who said they would not donate under any circumstances. To identify inconsistent responders, we compared responses to two related sets of questions.

The first set were:

- Let’s assume you were given the choice to donate your anonymous DNA information and medical information into a database AND it would only be accessed by medical doctors making a diagnosis in a patient. Whilst there might be benefits to patients from this work, medical doctors might benefit too. For example, through getting more diagnoses for patients and therefore being better at their jobs or getting scientific publications. Given the above assumptions, would you donate your anonymous DNA information and medical information for use by medical doctors?
- Let’s assume you were given the choice to donate your anonymous DNA information and medical information into a database AND it would only be accessed by non-profit researchers doing research, for example, on how DNA links to disease. There might be benefits to society from this work. But also, individual researchers and organisations might benefit too. For example, individual researchers could advance their career and organisations bring in new funding. Given the above assumptions, would you donate your anonymous DNA information and medical information for use by non-profit researchers?
- Let’s assume you were given the choice to donate your anonymous DNA information and medical information into a database AND it would only be accessed by for-profit researchers doing research, for example, developing new medicines. There might be benefits to society from this work. But also, individual researchers and organisations might benefit too. For example, individual researchers might advance their career and companies make a profit. Given the above assumptions, would you donate your anonymous DNA information and medical information for use by for-profit researchers?

The second question was:

Let’s assume you had the opportunity to donate your anonymous DNA information and medical information; to be used by any of the following:

- Medical doctors (e.g. to help them diagnose patients and also get scientific publications)
- Non-profit researchers (e.g. to do medical research and also bring in new funding)
- For-profit companies (e.g. to develop medicines and also make money for shareholders)

Within the above three scenarios, let’s also assume there is a chance that you can be *personally identified* by any of the researchers accessing your data.

Participants were asked to indicate whether or not they would donate, with those saying “yes” presented with a matrix of which type of researchers they would donate to versus the risk of being personally identified that they would accept. We deemed participants who said they would donate AND accept a risk of being identified in the second question, but had previously said that they would not consider donating at all, as being inconsistent responders. This was checked within each class of researcher (medical doctors, non-profit researchers, for-profit researchers). In total, 5.1% of participants gave inconsistent responses, and the percentage was similar across country subsamples. Additionally, two participants were removed because they did not answer the question about whether DNA information is different to other types of information. Removing these participants left a sample of 8,965 (3,316 from the UK; 1,992 from the United States; 2,255 from Canada; 1,402 from Australia).

**Supplemental References**

1. Van Gelder *et al*. Web-based questionnaires: The future in epidemiology? *Am J Epidemiol*. 2010;172(11):1292–8.
2. Frippiat *et al.* Web Surveys in the Social Sciences: An Overview. *Population-E*. 2010;65(2):285–311.
3. Barratt *et al.* Hidden Populations, Online Purposive Sampling, and External Validity: Taking off the Blindfold. *Field methods*. 2015;27(1):3.
